# Prevention of bone infection after open fracture using a chitosan with ciprofloxacin implant in animal model^1^

**DOI:** 10.1590/s0102-865020200080000003

**Published:** 2020-09-04

**Authors:** Silvia Iovine Kobata, Luiz Eduardo Moreira Teixeira, Simone Odilia Antunes Fernandes, André Augusto Gomes Faraco, Paula Vieira Teixeira Vidigal, Ivana Duval de Araújo

**Affiliations:** I Fellow Master degree, Postgraduate Program in Sciences Applied to Surgery and Ophthalmology Academic Unit, School of Medicine, Universidade Federal de Minas Gerais (UFMG), Belo Horizonte-MG, Brazil. Acquisition, analysis and interpretation of data; technical procedures; statistical analysis; manuscript preparation and writing.; II PhD, Full Professor, Locomotor System Department, Medical School, UFMG, Belo Horizonte-MG, Brazil. Critical revision, final approval.; III PhD, Associate Professor, Department of Pharmacy, UFMG, Belo Horizonte-MG, Brazil. Technical procedures, analysis and interpretation of data.; IV PhD, Associate Professor, Department of Pharmacy, UFMG, Belo Horizonte-MG, Brazil. Technical procedures;; V PhD, Full Professor, Histopathology Department, Medical School, UFMG, Belo Horizonte-MG, Brazil. Histopathological examinations.; VI PhD, Full Professor, Surgery Department, Medical School, UFMG, Belo Horizonte-MG, Brazil. Conception and design of the study, critical revision.

**Keywords:** Osteitis, Chitosan, Ciprofloxacin, Fractures, Open, Rats

## Abstract

**Purpose:**

To evaluate different concentrations of ciprofloxacin to prevent infection after open fracture contaminated with S. aureus in rats using absorbable local delivery system.

**Methods:**

Fifty-two Wistar rats were assigned to six groups. After 4 weeks, all animals underwent 99mTc-ceftizoxima scintigraphy evaluation, callus formation measurement and histological analysis. ANOVA, t-Student and Kruskal Wallis were used for quantitative variables statistical analysis, whereas qui square and exact Fisher were used for qualitative variables.

**Results:**

Treatment using 25% and 50% of ciprofloxacin incorporated at the fracture fixation device were effective in preventing bone infection compared to control group (p<0.05). Chitosan were not effective in preventing bone infection when used alone compared to control group (p>0.05). Histological findings demonstrated bone-healing delay with 50% of ciprofloxacin. No difference in callus formation were observed (p>0.05).

**Conclusion:**

Local delivery treatment for contaminated open fracture using chitosan with ciprofloxacin is effective above 25%.

## Introduction

Open fractures are mainly caused by high-energy traumatism with variable degree of soft tissue tears. Several consequences may result from open fracture – osteomyelitis is a feared complication that has proven difficult to treat especially after open fractures with Staphylococcal infection ^1 - 5^ . There are few reasons for difficulty of pathogen eradication like short life of antibiotics, poor blood circulation at the infected or necrotic area, degree of bacterial contamination, biofilm formation and systemic toxicity of antibiotics. To avoid infection, high doses of antibiotics at an open fracture site are necessary to ensure bioavailability, especially in Staphylococcal infections, whose biofilm growth mode shields them from systemic antibiotics ^6 - 13^ .

Despite all medical and pharmacological advances, infection rates after open fractures have not changed in the past 20 years, resulting in high social and economic costs. The pillars of infection prevention after open fractures have been mechanical debridement and systemic antibiotics ^1 - 8^ . However, previous experimental study in rats demonstrated that conventional systemic antibiotics could prevent infection in only 50% of cases, and that eradication of pathogen would be achieved only at a concentration 5 times higher than the conventional antibiotic dose ^14 - 16^ .

Controlled antibiotic release using a biodegradable system at open fracture contamination site could be a viable alternative for osseous infection prevention, minimizing systemic toxicity and enhancing optimal antibiotic concentration at contaminated site ^6 , 12 , 15 , 17 - 19^ .

In recent years, various biodegradable systems for local delivery of antibiotics have been studied. Chitosan is a natural hydrophilic polymer, versatile for its ability to carry drugs in a controlled delivery manner; it facilitates cellular regeneration and has some bactericide effect according to previous studies ^20 - 22^ .

Ciprofloxacin is a high-spectrum antibiotic used in daily practice because it has good bioavailability and osseous penetration, especially after rising of aminoglycosides bacterial resistance.

The rationale for appropriate antimicrobial treatment depends not only on the spectrum of antibiotic activity, but also on the optimal concentration at infection site. Several systemic treatments intended to favor more sustained concentration at plasma were tested ^1 , 5 , 6^ . However, there is a lack of information on which concentration of antibiotic-loaded using absorbable delivery system could, in fact, be efficient for the infection control without deleterious problems of antibiotic toxicity in high doses.

The objective of this study is to determine what concentration of ciprofloxacin would be efficient for infection control in rats, after open fracture contaminated by *Staphylococcus aureus* , using chitosan as an absorbable delivery system.

## Methods

All experiments were carried out in accordance with the Animal Welfare Committee. The Ethical Committee of Animal Experimentation (CONCEA) approved the research under study protocol (nº 228/2014 in 24/09/2014). The experiments were conducted in the Laboratory of Experimental Surgery of the Universidade Federal de Minas Gerais (UFMG) and scintigraphy analysis was conducted in the Radioisotope Laboratory of Pharmacy of UFMG.

### *Animals*

Fifty-two male adult Wistar rats (Rattus norvegicus albinus, Rodentia mammalia) at least 3 months old and weighing about 300 g (range 301-325 g) were selected from Federal University of Minas Gerais laboratory. The rats were housed in a constant room temperature and fed standard laboratory diet. After sample calculation with 90% of power and 5% degrees of significance, the study was designed with 52 animals divided into six groups.

All animals underwent open fracture model procedure and were contaminated with Staphylococcus aureus. After contamination, the animals were randomly divided into six groups according to protocol of treatment. Groups of animals:

– Control Group. Contaminated open fracture treated with metallic rod osteo-fixation without antibiotics (n=11).
– Treatment control. Contaminated open fracture treated with metallic rod osteo-fixation and systemic ciprofloxacin (20mg/kg) for 72 hours (n=8).
– Carrier control. Contaminated open fracture treated with chitosan impregnated in the metallic rod used for osteo-fixation without antibiotics (n=8).
– Ciprofloxacin at 10%. Contaminated open fracture treated with chitosan impregnated with 10% ciprofloxacin in the metallic rod used for osteo-fixation (n=7).
– Ciprofloxacin at 25%. Contaminated open fracture treated with chitosan impregnated with 25% ciprofloxacin in the metallic rod used for osteo-fixation (n=8).
– Ciprofloxacin at 50%. Contaminated open fracture treated with chitosan impregnated with 50% ciprofloxacin in the metallic rod used for osteo-fixation (n=10).

### *Preparation of implants*

Cylindrical titanium K-wire implants (1.5 mm of diameter and 60 mm of length) sterilized and impregnated with chitosan and ciprofloxacin in different concentrations (0%, 10%, 25% and 50%) were prepared according to the protocol of Laboratory of Pharmacy at UFMG as follows ^23^ .

For each K-wire rod, a 2.5% p/v mixture of chitosan polymer (25 mg of chitosan) with ciprofloxacin incorporated according to the following protocol: ciprofloxacin 10% - impregnated with 2.5 mg ciprofloxacin; ciprofloxacin 25% - impregnated with 6.75 mg ciprofloxacin; and ciprofloxacin 50% - impregnated with 12.5mg ciprofloxacin. All impregnated rods were maintained under controlled temperature and pH until surgical procedure was executed in the next 24 hours of preparation.

### *Preparation of bacterial suspension and inoculum standardization*

Methicillin-sensitive *Staphylococcus aureus* from stock cultures (ATCC6538-P) were selected and cultivated in agar media. Subsequent to incubation for 18-24 hours at 32 ± 2°C, colonies had concentrations adjusted to 1x10 ^9^ CFU/ml using spectrophotometer analysis and 1ml of homogenized bacterial suspension with 9 ml of trypticase soy agar spread into Petri dish of 80cm of diameter. Rectangles of 5 x 2.5cm corresponding to 2.6 x 10 ^6^ CFU were extracted and used as animal inoculum.

### *Surgical procedure*

**Figure 1:**
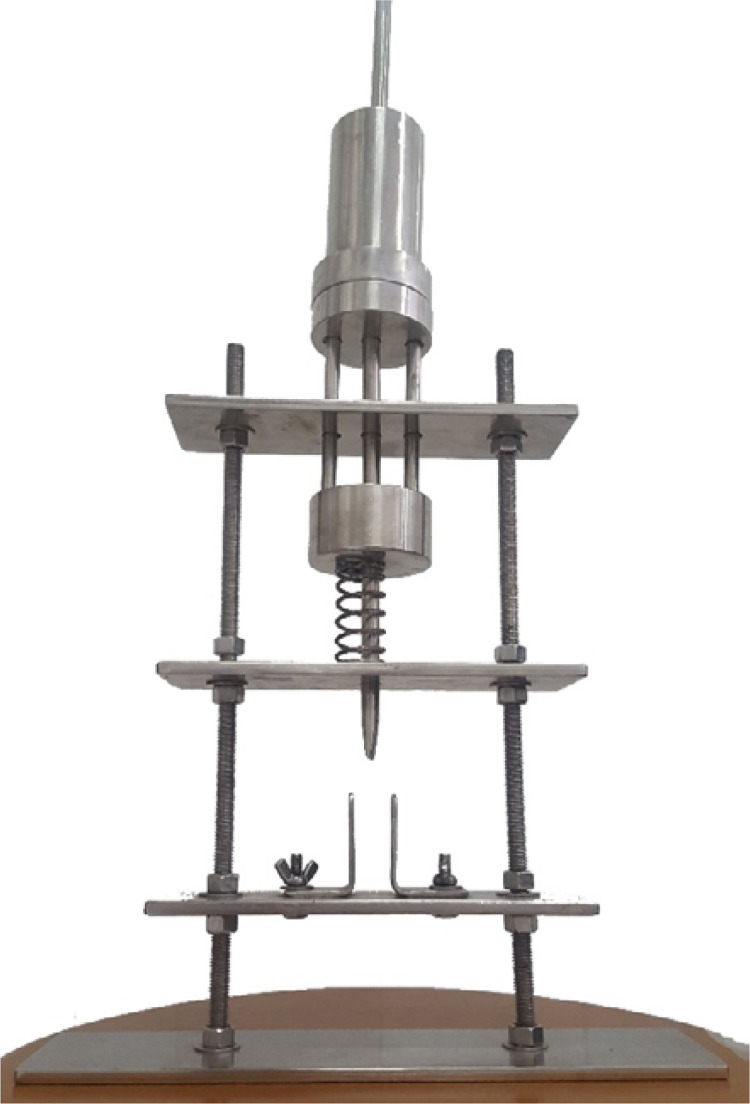
Guillotine device for closed and standardized diaphyseal fracture.

For surgical procedure, the animals were anesthetized and maintained under sedation by intraperitoneal injection with ketamine and xylazine (15 mg/kg and 60 mg/kg, respectively). All animals underwent closed diaphyseal femoral fracture using a guillotine equipment that reproduces similar transverse pattern fracture in all animals (Fig. 1). After trichotomy and sterilization with polyvinylpyrrolidone (Povidine® - Johnson & Johnson, Brazil), the skin was incised, the femoral fracture was contaminated with 2.6 x 10 ^6^ UFC of Staphylococcus aureus and left open for one hour. After 1 hour of contamination, open reduction and intramedullary ﬁxation were performed using a 1.0 K-wire and skin was closed by stiches. Group B animals had their first systemic dose of ciprofloxacin right after surgical procedure.

### *Scintigraphy*

Four weeks after surgery, all animals underwent antibiotic marked scintigraphy exam under anesthesia, and images were obtained after 360 minutes of ceftizoxime-technetium injection according to Teixeira *et al* . ^23^ .

Ceftizoxime (Ceftizox® SmithKline Beecham, Rio de Janeiro, Brazil) dissolved in a 1.2 ml solution containing pertechnetate (Na ^99m^ TcO _4_ ) and heated for 100 ^0^ for 10 minutes. The reaction mixture was then cooled in running water for 5 minutes and was radiopharmaceutically collected after vacuum filtration through a 0.22 mcm cellulose membrane.

After 360 minutes of radiopharmaceutical injection in the animals under anesthesia, they were then positioned in dorsal decubitus and static planar images were obtained using a 256x256 pixel matrix (Nuclide ^TM^ TH22, Mediso ^R^ , Hungary) after 10 minutes of radioactivity count in each femur “region of interest” (ROI) for residual infection analysis detection.

### *Histological analysis*

After sacrificing animals, all femurs collected had macroscopic and histologic analyses performed. The fracture site was studied for the presence of intraosseous abscess and osseous callus when present, measured in millimeters.

For histological examination, all samples were stained with hematoxylin-eosin and evaluated by the same blinded-pathologist using conventional light microscopy (Olympus AX70 microscope™, German) searching for osteomyelitis activity. Lesion analysis was performed in 5 adjacent high power field (HPF) areas for each animal sample. Histological parameters were standardized as intraosseous acute inﬂammation (inflammatory cells infiltration), intraosseous chronic inﬂammation (angiogenesis), periosteal inﬂammation (matrix formation) and bone necrosis ^23 - 25^ . Therefore, each parameter was graded, classified and divided into two groups: mild or intense. For angiogenesis, mild had less than 5 neo formed vessels found in each HPF and intense had more than 5 neo formed vessels in each HPF. For acute inflammation, mild degree had sparse neutrophils surrounded by regular tissue, and intense had aggregates of neutrophils in any field. For matrix formation, mild had absence of any thick connective tissue, and intense had osseous matrix and thick collagen encountered without soft connective tissue surrounding. Osseous trabeculae without infection lamina were utilized as control for reading standardization purposes.

### *Statistical analysis*

Statistical analysis was performed utilizing SPSS ^TM^ (IBM Statistical Package of Social Science software, USA). Shapiro_Wilk test was utilized for normality analysis. Chi square with Fischer correction was utilized for qualitative data calculation, and ANOVA, t-Student and Kruskal Wallis for quantitative data analysis. Statistical difference was considered when p value was inferior to 0.05.

## Results

All 52 animals survived the treatment. During macroscopic analysis, seven animals presented periosteal abscess (13.46%). Interestingly, none of the animals from groups B and F presented any sign of macroscopic suppuration at fracture site (  Table 1  ).

**Table 1:** Frequency of wound suppuration after 4 weeks of open fracture with *Staphylococcus aureus* contamination in rats.

Groups (n)	Suppuration	
	n	%
A (n = 11)	3	27.0
B (n = 8)	0	0
C (n = 8)	2	25.0
D (n = 7)	1	14.0
E (n = 8)	1	12.5
F (n = 10)	0	0
TOTAL (n = 52)	7	13.5

*p* =0.3591; A – no treatment; B – Systemic ciprofloxacin; C – Chitosan impregnated rod; D – Chitosan impregnated rod with 10% ciprofloxacin; E – Chitosan impregnated rod with 25% ciprofloxacin; F – Chitosan impregnated rod with 50% ciprofloxacin.

**Figure 2:**
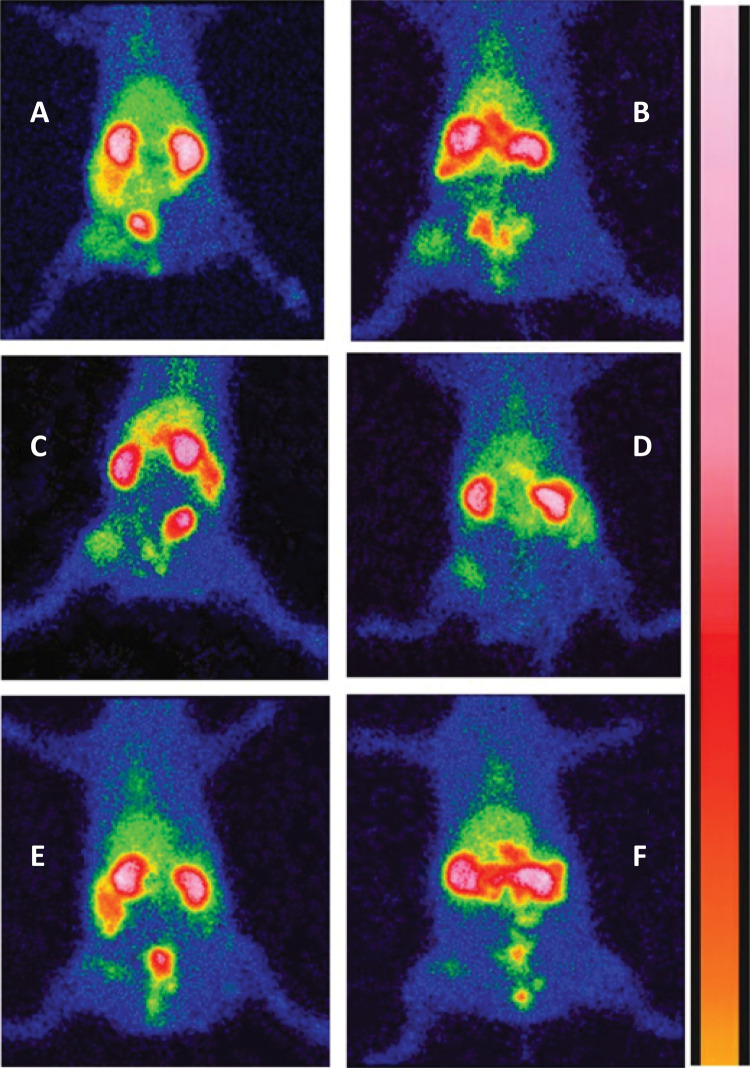
Scintigraphy after 6 hours injection of 99mTc-CFT demonstrating different concentrations in the right thigh 4 weeks after S. aureus contaminated open fracture, according to each animal group of treatment. A – No treatment; B – Systemic ciprofloxacin; C – Chitosan impregnated rod; D – Chitosan impregnated rod with 10% ciprofloxacin; E – Chitosan impregnated rod with 25% ciprofloxacin; F – Chitosan impregnated rod with 50% ciprofloxacin.

Callus measurement in centimeters according to each group ranged 0.3845± 0.3896 in group A; 0.7013 ± 0.5191 in group B; 0.5675 ± 0.39507 in group C; 0.3943 ± 0.54659 in group D; 2.38 ± 4.55015 in group E, and 4.2990 ± 5.91925 in group F. Despite the higher apparent callus formation in groups E and F there was no statistical difference between groups (p=0.088) (Fig. 2).

**Figure 3:**
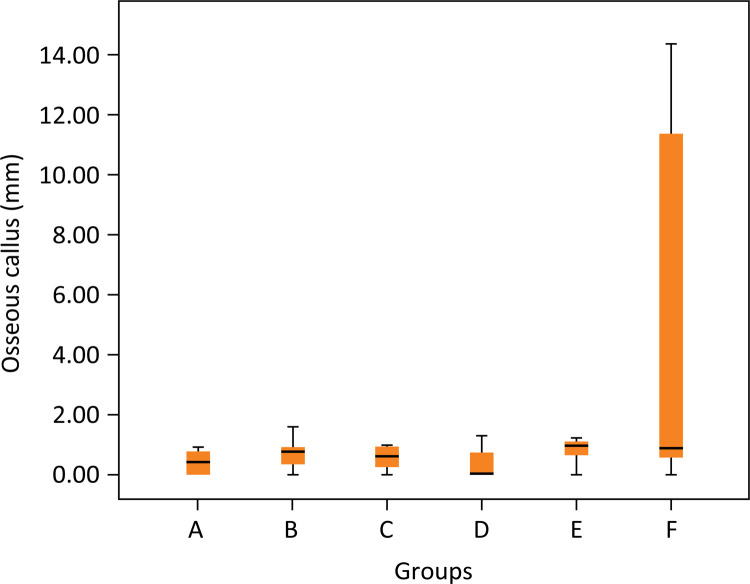
Macroscopic osseous callus measurement after 4 weeks of open fracture with Staphylococcus aureus contamination in rats according to each group of treatment (p>0.05). Groups: A – No treatment; B – Systemic ciprofloxacin; C – Chitosan impregnated rod; D – Chitosan impregnated rod with 10% ciprofloxacin; E – Chitosan impregnated rod with 25% ciprofloxacin; F – Chitosan impregnated rod with 50% ciprofloxacin.

**Figure 4:**
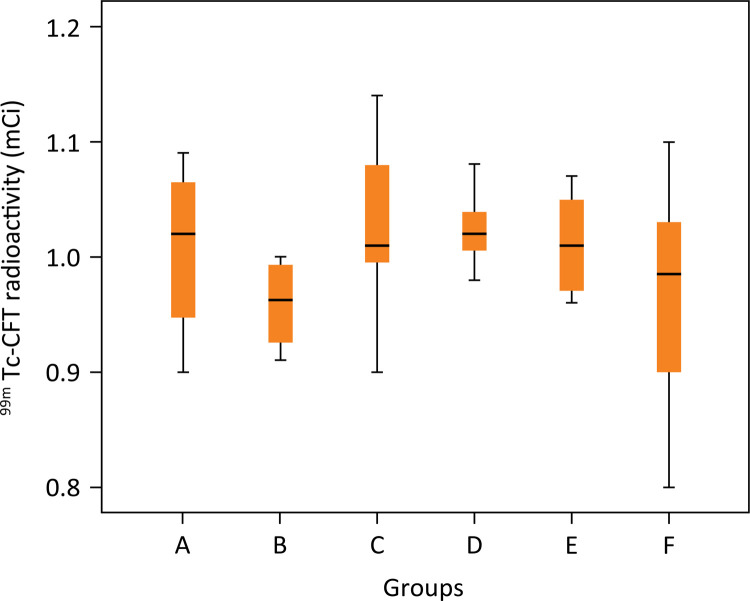
99mTc-CFT Radioactivity analysis before injection in animals (p>0.05). Groups: A – No treatment; B – Systemic ciprofloxacin; C – Chitosan impregnated rod; D – Chitosan impregnated rod with 10% ciprofloxacin; E – Chitosan impregnated rod with 25% ciprofloxacin; F – Chitosan impregnated rod with 50% ciprofloxacin.

The scintigraphy analysis demonstrated 93% purity of Ceftizoxime-technetium ( ^99m^ Tc-CFT) in radiopharmaceutical evaluation in the previous injection analysis. There was no statistical difference between radioactivity of ^99m^ Tc-CFT samples before injection into the animal (p=0.177) (Fig. 3). There was no statistical difference between the residual radioactivity of each syringe analysis after injection into the animal (p=0.144) (Fig. 4).

**Figure 5:**
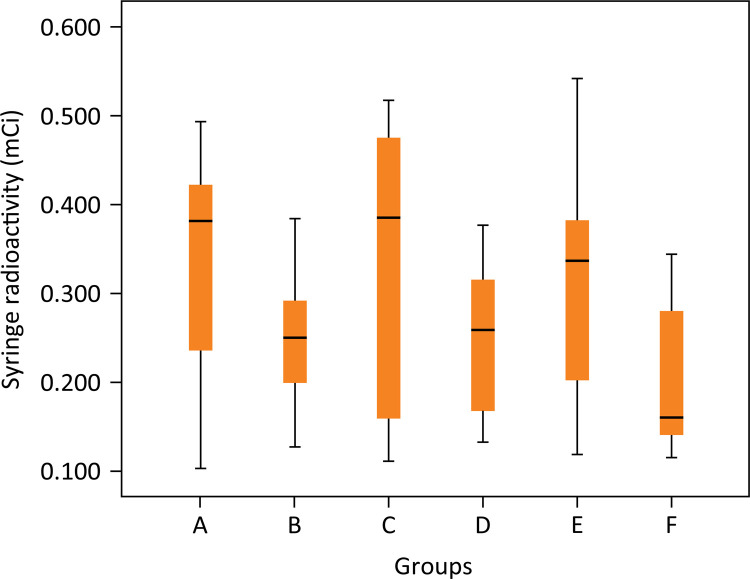
Residual radioactivity measurement of the syringe utilized in each animal group. (p>0.05). Groups: A – No treatment; B – Systemic ciprofloxacin; C – Chitosan impregnated rod; D – Chitosan impregnated rod with 10% ciprofloxacin; E – Chitosan impregnated rod with 25% ciprofloxacin; F – Chitosan impregnated rod with 50% ciprofloxacin.

After injection, all the animals presented a high concentration of radiation in the urinary tract and in the infected femur (ROI), demonstrating depuration of the radiopharmaceutical in the urinary tract and high affinity of Ceftizoxime-technetium only with the infection site (Fig. 5).

**Figure 6:**
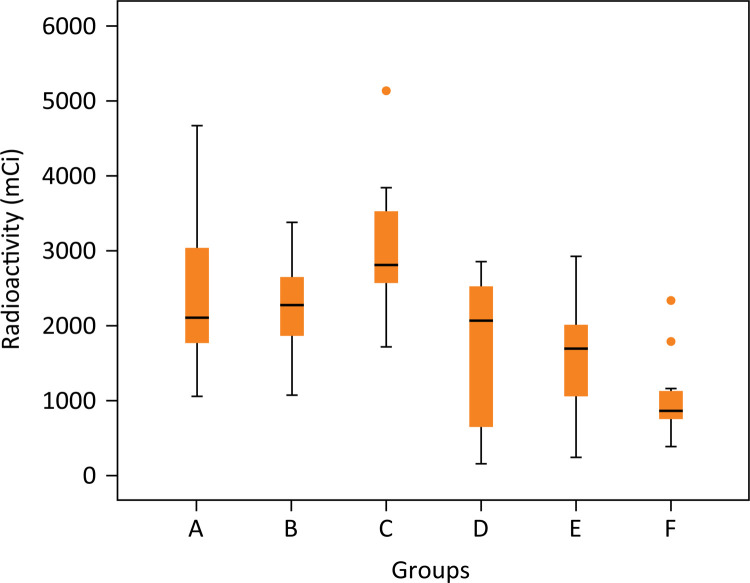
Radioactivity analysis of residual infection in the animal thigh after 4 weeks of open fracture in rats with S aureus contamination demonstrating effect of different treatment group. (p>0.05). Groups: A – No treatment; B – Systemic ciprofloxacin; C – Chitosan impregnated rod; D – Chitosan impregnated rod with 10% ciprofloxacin; E – Chitosan impregnated rod with 25% ciprofloxacin; F – Chitosan impregnated rod with 50% ciprofloxacin.

**Figure 7:**
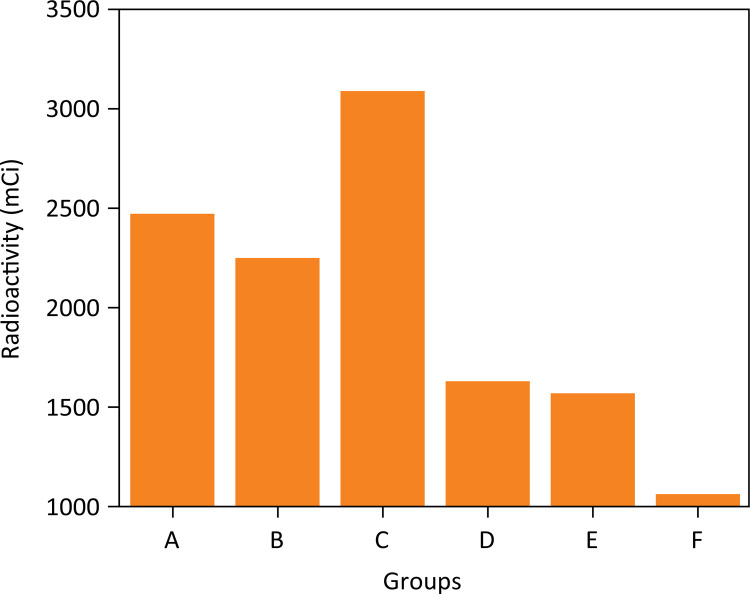
Radioactivity measurement in the animal thigh after 4 weeks of open fracture with Staphylococcus aureus contamination in rats according to each group of treatment. (p>0.05). Groups: A – No treatment; B – Systemic ciprofloxacin; C – Chitosan impregnated rod; D – Chitosan impregnated rod with 10% ciprofloxacin; E – Chitosan impregnated rod with 25% ciprofloxacin; F – Chitosan impregnated rod with 50% ciprofloxacin.

Values obtained after scintigraphy analysis ranged from 163 to 5152 at the ROI (SD=1109, mean value=2010). The scintigraphy values obtained according to each group were:2468.0 ± 1127.550 in group A, 2258.0 ± 698.733 in group B, 3094.0 ± 1031.037 in group C, 1633.57 ± 2055.0 in group D, 1574.5 ± 832.736 in group E, and 1057.3 ± 587.013 in group F (Figs. 6 and 7).

Statistical differences were found between groups A and F (p=0.015), groups C and E (p=0.028) and between groups C and F (p=0.000) (  Table 2  ).

**Table 2:** Scintigraphy with radioactivity measurement in the animal thigh after 4 weeks of open fracture with *Staphylococcus aureus* contamination in rats according to each group of treatment.

Groups (n)	Scintigraphy	
	Mean	Standard deviation
A (n =11)	2468.0	1127.6
B (n =8)	2258.0	698.7
C (n =8)	3094.0	1031.0
D (n =7)	1633.6	1119.2
E (n =8)	1574.5	832.7
F (n =10)	1057.3	587.0

*P* <0.05; Groups: A – no treatment; B – Systemic ciprofloxacin; C – Chitosan impregnated rod; D – Chitosan impregnated rod with 10% ciprofloxacin; E – Chitosan impregnated rod with 25% ciprofloxacin; F – Chitosan impregnated rod with 50% ciprofloxacin

During the histological analysis no statistical difference was found between the groups for inflammatory cells (p=0.675), angiogenesis (p=0.802) or fibroplasia (p>0.05) (Tables 3 [Table t4] to 5).

**Table 3:** Histological evaluation of inflammatory cell infiltration after 4 weeks of open fracture with *Staphylococcus aureus* contamination in rats according to each group.

Groups (n=52)	Inflammatory cell			
	Mild		Intense	
	N	%	n	%
A (n =11)	8	72.7	3	27.3
B (n =8)	7	87.5	1	12.5
C (n =8)	5	62.5	3	37.5
D (n =7)	4	57.1	3	42.9
E (n =8)	4	50.0	4	50.0
F (n =10)	7	70.0	3	30.0

*p* =0.675; Groups: A – no treatment; B – Systemic ciprofloxacin; C – Chitosan impregnated rod; D – Chitosan impregnated rod with 10% ciprofloxacin; E – Chitosan impregnated rod with 25% ciprofloxacin; F – Chitosan impregnated rod with 50% ciprofloxacin.

**Table 4 t4:** Histological evaluation of angiogenesis after 4 weeks of open fracture with *Staphylococcus aureus* contamination in rats according to each group.

Groups (n =52)	Angiogenesis			
	Mild		Intense	
	N	%	N	%
A (n= 11)	9	81.8	2	18.2
B (n= 8)	4	50.0	4	50.0
C (n= 8)	5	62.5	3	37.5
D (n= 7)	5	71.4	2	28.6
E (n= 8)	5	62.5	3	37.5
F (n= 10)	6	60.0	4	40.0

*p* = 0.802; Groups: A – no treatment; B – Systemic ciprofloxacin; C – Chitosan impregnated rod; D – Chitosan impregnated rod with 10% ciprofloxacin; E – Chitosan impregnated rod with 25% ciprofloxacin; F – Chitosan impregnated rod with 50% ciprofloxacin.

**Table 5:** Histological evaluation of fibroplasia (conjunctive dense tissue) after 4 weeks of open fracture with *Staphylococcus aureus* contamination in rats according to each group.

Groups (n =52)	Fibroplasia			
	Mild		Intense	
	N	%	n	%
A (n =11)	5	45.5	6	54.5
B (n =8)	7	87.5	1	12.5
C (n =8)	6	75.0	2	25.0
D (n =7)	3	42.9	4	57.1
E (n =8)	5	65.2	3	37.5
F (n =10)	5	50.0	5	50.0

*p* >0.05; Groups: A – no treatment; B – Systemic ciprofloxacin; C – Chitosan impregnated rod; D – Chitosan impregnated rod with 10% ciprofloxacin; E – Chitosan impregnated rod with 25% ciprofloxacin; F – Chitosan impregnated rod with 50% ciprofloxacin.

There was a statistical difference between groups A and F when osseous matrix formation was studied (p=0.023). Group A had a predominant intense degree osseous matrix formation and group F had a predominant mild osseous matrix formation (  Table 6  ).

**Table 6:** Histological evaluation of osteogenic matrix after 4 weeks of open fracture with *Staphylococcus aureus* contamination in rats according to each group.

Groups (n =52)	Osteo Matrix			
	Mild		Intense	
	N	%	n	%
A (n =11)	4	36.4	7	63.6
B (n =8)	7	87.5	1	12.5
C (n =8)	6	75.0	2	25.0
D (n =7)	4	57.1	3	42.9
E (n =8)	6	75.0	2	25.0
F (n =10)	10	100.0	0	0.0

*p* <0.05; Groups: A – no treatment; B – Systemic ciprofloxacin; C – Chitosan impregnated rod; D – Chitosan impregnated rod with 10% ciprofloxacin; E – Chitosan impregnated rod with 25% ciprofloxacin; F – Chitosan impregnated rod with 50% ciprofloxacin.

## Discussion

Deep bone infection after contaminated open fracture is a serious complication; attempts to prevent it are based on early surgical debridement and large-scale systemic antibiotics use. Despite all medical and pharmacological advances, infection rates after open fractures have not changed in the past 20 years, promoting high social and economic costs. Over the past decades, several attempts have been made to prevent osteomyelitis occurrence by incorporating antibiotics into local delivery systems to reduce osseous infections rates. However, which concentration of antibiotic releasing system would have clinical efficacy is still controversial.

On the one hand, the presence of high doses of antibiotics in a local delivery system can lead to toxicity symptoms and impairment of tissue healing; on the other hand, the presence of low dose of antibiotics over an extended period at any infection site can enhance antibiotic resistance, so feared in medicine today.

The objective of this study was to determine which concentration of ciprofloxacin, commonly used as first line of antibiotics in different orthopedics infections situations, would in fact be efficient for infection control after open fracture contaminated with S aureus using chitosan as an absorbable delivery system in rats (10%, 25% or 50% ciprofloxacin concentrations). A bioabsorbable delivery system allows all impregnated antibiotics to be available at the infection site in contrast to cement-based delivery system, where only 10-20% of impregnated antibiotics are actually available at infection site.

Although previous studies have described chitosan having antibiotic properties, in our study chitosan was not effective in preventing bone infection when used alone compared to control group (p>0.05).

Traditional tecnecium-99 scintigraphy offers high sensibility and low specificity in the detection of acute osseous infections. However, use of ceftizoxime labeled with metastable isomer of technetium-99 ( ^99m^ Tc-ceftizoxime) improved specificity by comparing aseptic osseous inflammation with septic osseous inflammation in previous studies ^4 , 25^ .

Scintigraphy analysis demonstrated 93% purity of Ceftizoxime-technetium ( ^99m^ Tc-CFT) in radiopharmaceutical evaluation in previous injection analysis. After injection, all animals presented high concentration of radiation only in the urinary tract and in the infected femur (interest site), demonstrating depuration of the radiopharmaceutical in the urinary tract and high affinity of the Ceftizoxime-technetium only with the infection site according to previous studies.

There was no statistical difference between radioactivity of ^99m^ Tc-CFT samples before injection into the animal (p=0.177) or residual radioactivity of each syringe analysis used after injection into the animal (p=0.144). In conclusion, the same amount of radioactivity was bioavailable in all subjects.

The scintigraphy study demonstrated that treatment using 25% and 50% of ciprofloxacin incorporated in the fracture fixation device was effective in preventing bone infection compared to the control group (p<0.05). However, the use of 10% ciprofloxacin was not effective in preventing bone infection, according to the previous study ^26^ .

There was no statistical difference between the groups treated with systemic antibiotics and the control group (p>0.05), demonstrating low efficacy of systemic treatment as an isolated procedure after contaminated open fractures. These findings corroborate the study by Greenberg et al that described rates of 60% of remaining infection after ciprofloxacin systemic treatment for *Staphylococcus aureus* osteomyelitis. Similar findings were also encountered in animal models studies by Paiva Costa *et al* . ^26^ , and Penn-Barwell *et al* . ^27^ that encountered no infection control after systemic ciprofloxacin as an isolated treatment for osseous *Staphylococcus aureus* infection in rats.

During macroscopic analysis, seven animals presented periosteal abscess (13.46%). Interestingly, none of the animals from groups B and F presented any sign of macroscopic suppuration at fracture site, which demonstrates efficient infection control with systemic antibiotics and high doses of local use of ciprofloxacin (50%).

Callus measurement in centimeters according to each group ranged 0.3845± 0.3896 in group A; 0.7013 ± 0.5191 in group B; 0.5675 ± 0.39507 in group C; 0.3943 ± 0.54659 in group D; 2.38 ± 4.55015 in group E and 4.2990 ± 5.91925 in group F. Despite the higher apparent callus formation in animals treated with 25% and 50% ciprofloxacin, there was no statistical difference between the groups (p=0.088) in accordance with previous studies that demonstrated that the use of ciprofloxacin in the local delivery system would not impair osseous consolidation rates ^1 , 22 , 28 , 29^ .

Histological findings demonstrated bone-healing delay and high variability of size callus formation only in the treatment with 50% ciprofloxacin. However, no difference in callus formation measurement was observed between the groups (p>0.05). These findings could be attributed by higher heterogeneity callus formation after 50% of ciprofloxacin. Similar findings were described in a study by Kupczik *et al* . ^24^ , who showed that high doses of ciprofloxacin could interfere with matrix formation with no difference in callus mechanical resistance, and supported by Miclau *et al* . ^30^ , that demonstrated decrease of osteoblasts number in a dose dependent manner when the osteoblasts were exposed to high concentration of ciprofloxacin.

In this study we could infer that, despite chronic infection, osseous tissues are still capable to regenerate properly, and high doses of antibiotics could interfere in matrix formation in cellular level, but macroscopic callus formation may not present significant alteration in rats.

This study demonstrated that the bioabsorbable polymer impregnated with ciprofloxacin is effective in preventing infection in rats after open fracture contamination with *S aureus* , when the ciprofloxacin concentration is above 25%. However, clinicians may be aware of potentially adverse effects of high levels of antibiotics with local application and avoid minimal therapeutic level that can induce bacterial resistance.

Therefore, further in vivo studies are needed to understand what dose of antibiotics used in the local delivery system should be optimal in clinical practice in humans.

## Conclusion

Local delivery treatment for contaminated open fracture with *Staphylococcus aureus* using a bioabsorbable polymer impregnated with ciprofloxacin is effective in rats when ciprofloxacin concentration is above 25%.

## Footnotes

1 Research performed at Laboratory of Experimental Surgery, and Radioisotope Laboratory of Pharmacy, Universidade Federal de Minas Gerais (UFMG), Belo Horizonte-MG, Brazil. Part of Master degree thesis, Postgraduate Program in Sciences Applied to Surgery and Ophthalmology Academic Unit. Tutor: Profa Ivana Duval de Araújo.
